# Long-term survival without high cancer risk in a cohort of 24 patients with Apert syndrome

**DOI:** 10.1038/s41431-026-02122-w

**Published:** 2026-05-04

**Authors:** Benjamin J. Cairns, Dominique M. Davidson, Sarah F. Smithson, Andrew O. M. Wilkie

**Affiliations:** 1 Our Future Health, London, UK; 2 Edinburgh Department of Plastic Surgery, Lothian, UK; 3 Department of Clinical Genetics, University Hospitals Bristol and Weston NHS Foundation Trust, Bristol, UK; 4 MRC Weatherall Institute of Molecular Medicine, University of Oxford, Oxford, UK

**Keywords:** Disease genetics, Cancer

## Abstract

The classification of congenital malformations has been transformed over recent decades by advances in genetic analysis, so that the natural history of many disorders during childhood is well described. However, implications for adult prognosis and survival are often poorly documented. In Apert syndrome, caused by heterozygous germline mutations in the fibroblast growth factor receptor type 2 gene (*FGFR2*), the question of prognosis is particularly pertinent because *FGFR2* is a known cancer driver gene (oncogene) and the identical mutations, when arising somatically, are enriched in specific tumours, notably endometrial carcinoma. We exploited a unique resource provided by a series of 24 UK patients described by Dr Eric Blank in 1960, and used tracing of cancer events and deaths through the National Health Service Central Register to determine the long-term outcome of these individuals until 2013, a period spanning 53 years. Twelve individuals (50%) were still alive and without any cancer registration, at the end of the study; of the remainder, two could not be traced and ten were known to have died, with four deaths related to malignancies. We conclude that Apert syndrome is not, in many affected individuals, associated either with substantial shortening of lifespan, or with a high risk of developing particular types of cancer. Explanation of the lack of strong cancer predisposition, despite the oncogenic nature of the *FGFR2* mutations, may lie in the different signalling relationship that a mutant cell has with its neighbours when the mutation is present constitutionally, compared to occurrence as a somatic change.

## Introduction

Apert syndrome is a classic, relatively common malformation syndrome (birth prevalence ~1 in 70,000) [1, 2], that is usually easily diagnosed clinically, based on the characteristic combination of complex craniosynostosis affecting multiple sutures of the skull and complete syndactyly involving at least the central three digits of the hands and feet [3]. The disorder is named after Eugène Apert, who described a series of eight patients in 1906 [4], and was recognised to have a genetic basis when the first parent-child transmission was reported [5]; however most cases occur sporadically.

In 1960 Eric Blank, a British geneticist, published a series of 39 unrelated singleton cases who exhibited the pathognomonic clinical features of Apert syndrome; this was the largest series in the world at the time, and highlighted an increase in the unaffected parents’ mean age, which could be wholly attributed to the contribution of the father’s age (paternal age effect) [6]. In 1995 it was demonstrated that Apert syndrome is caused, in ~99% of cases, by one or other of two heterozygous nucleotide substitutions in the *FGFR2* gene [NM_000141.5:c.755 C > G encoding p.(Ser252Trp) or NM_000141.5:c.758 C > G encoding p.(Pro253Arg)] [7]; the de novo mutations locate exclusively on the allele transmitted from the unaffected father [8]. Subsequent work has shown that the amino acid substitutions activate the receptor by increasing the affinity, and broadening the specificity, of ligand binding (reviewed in [9]); the enhanced ligand-dependent receptor activation promotes overactive signalling through the downstream RAS-MAP kinase-ERK pathway, a key regulator of cell behaviour [10, 11]. When such a mutation arises in the adult testis, it leads to clonal expansion of mutant cells [12, 13], a process termed selfish spermatogonial selection. This accounts for the high apparent mutation rate and paternal age effect in Apert syndrome (reviewed in [14]).

Owing to the characteristic presentation, limited mutational spectrum and relatively high prevalence of Apert syndrome, the clinical features and complications in affected children have been comprehensively catalogued [15, 16]. However, whether there are additional medical problems in adulthood is less well documented. Several studies report the follow-up of individuals with Apert syndrome into their 30s [17–19] and an individual aged 73 years was described in a recent case report [20], but none has systematically ascertained individuals at older ages. A particularly important unanswered biological question is whether Apert syndrome may confer susceptibility to certain cancers. Although *FGFR2* is not a recognised cancer predisposition gene [21] there are several reports of rare tumours occurring in children with Apert syndrome [22–25]. Moreover *FGFR2* is a well-established cancer driver gene (oncogene) [26–28]. According to the COSMIC database v103, released 18/11/2025 [29], somatic *FGFR2* missense mutations are particularly frequent in cancers involving the endometrium, liver, meninges and skin, occurring in 5–12% of cases involving each of these tissues. Notably, the mutation encoding p.(Ser252Trp) (identical to that occurring constitutionally in Apert syndrome) is the most prevalent hotspot substitution in cancer over the entire FGFR2 protein (*n* = 191 representing 13.4% of all *FGFR2* missense substitutions; 84% of p.(Ser252Trp) cases were identified in endometrial cancers). p.(Pro253Arg), the other substitution equivalent to that commonly occurring in Apert syndrome, is less frequently observed in cancers (*n* = 31 representing 2.2% of all *FGFR2* missense mutations, of which 52% were in endometrial cancers) [29]. Given the demonstrated effect of these mutations in activating the RAS-MAP kinase-ERK pathway [10], it is pertinent that five components of this pathway (NF1, HRAS, KRAS, PTPN11/SHP2, SOS1) are encoded by proven cancer predisposition genes [21], all of which (except *NF1*) act through gain-of-function mechanisms to predispose to a variety of malignancies in children and young adults harbouring constitutional mutations (so-called RASopathies). Hence it is plausible that *FGFR2* might also act as a cancer predisposition gene, but solid clinical evidence beyond statistical association of common non-coding variants that have low effect sizes [30] is so far lacking.

Here we explore whether individuals with Apert syndrome have increased mortality from cancer or other causes, by presenting the first long-term study of survival of Apert syndrome patients into later adulthood. Using the details provided by Dr Blank on the 24 patients still alive at the time of his study [6], we have been able to trace the outcomes of all except two of these individuals over more than half a century.

## Methods

This study was initiated in 1994 as part of a research programme to identify the genetic basis of Apert syndrome. Ethical permission to perform this work was provided by the Central Oxford Research Ethics Committee (COREC 2742) and the protocol was approved by the Medical Research Unit of the Office of Population Censuses & Surveys (OPCS), reference MR 461. The British Medical Association Ethics Committee approved the provision of cancer registration data. We contacted Dr Eric Blank (Sheffield), who kindly provided the names, dates of birth and historical addresses of 24 individuals (including 23 personally examined by him) alive in 1958–1959 and confirmed to have Apert syndrome, based on the characteristic combination of clinical features as summarised in his publication [6]. The birth years of these subjects ranged from 1911–1958 (mean 1940, median 1944, corresponding to a median of ~15 years of age at the time of ascertainment). This information was passed to OPCS (later renamed the National Health Service [NHS] Central Register and subsequently the NHS Information Centre) for tracing either the death certificate, or the current address and contact details of the General Practitioner (GP). All but one of the individuals traced resided in England or Wales. In the case of 16 individuals traced and still alive, we wrote to the GP requesting permission to contact the patient, and if this was granted, we wrote to the patient requesting permission to perform a home visit. Five individuals gave this permission, were examined in 1996–1997 and the clinical and molecular diagnosis of Apert syndrome was confirmed in all cases (3 had the *FGFR2* NM_000141.5:c.755 C > G and 2 the NM_000141.5:c.758 C > G mutation; these results were included in a previous molecular analysis of Apert syndrome [31]).

**Fig. 1:**
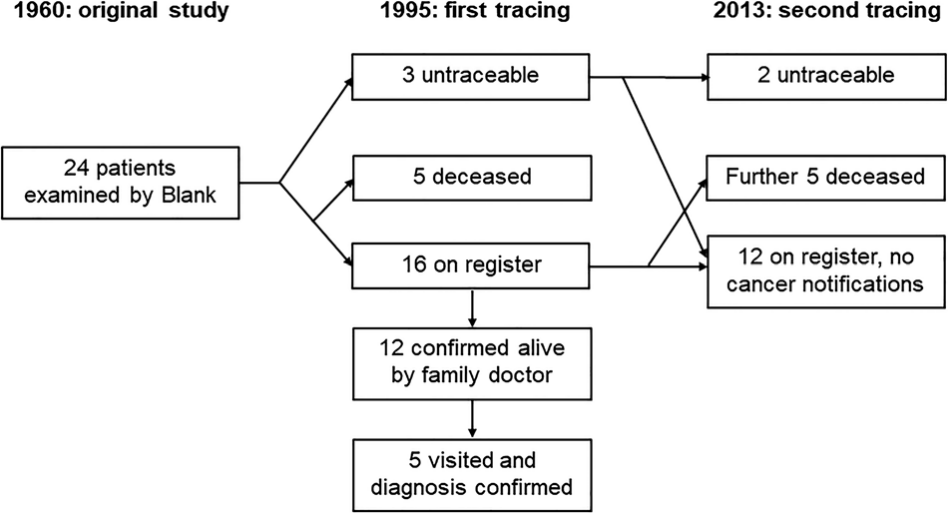
Flow chart summarising the course of the study. Boxes classify the patients into different groups, starting (at left) with Blank’s 1960 publication [6] and finishing (at right) in 2013. Lines with arrows connect the groups at different timepoints.

Notification of cancer registrations and deaths continued over subsequent years. In 2012, by which time a total of 10 deaths had been recorded, a second attempt to trace the other 14 individuals was made. This indicated that 12 were on the NHS register and presumed alive and without cancer registrations, and 2 remained untraceable. The status of the 24 subjects in May 2013, when the study was closed, is summarised in Fig. 1: at this timepoint, 12 subjects were still alive, 10 had died, and 2 were untraceable. Following changes in UK legislation (General Data Protection Regulation), a Data Sharing Agreement was approved by NHS Digital in 2019 to retain the data following pseudonymisation by removal of names and rounding days of birth and death to the first day of the month (current ref. DARS-NIC-148106-PP9LS-v.4.4).

Age-specific cancer and all-cause mortality rates were estimated for the general population of England and Wales from the WHO Mortality Database [32]. Aggregate expected numbers of deaths (either from all causes or from cancer specifically) were generated from these mortality rates for the time at risk of the Apert syndrome cohort between 1959 and 2013. The observed numbers of deaths in the cohort were divided by the expected numbers of deaths to calculate standardised mortality ratios (SMRs) with exact Poisson 95% confidence intervals (treating the population mortality rates as fixed). To illustrate mortality over time, Kaplan-Meier survival curves were generated and plotted for both all-cause and cancer-specific mortality.

## Results and discussion

Over a 54-year period (1959–2013), follow-up of 12 out of the 24 individuals with Apert syndrome ended, either because the person died (*n* = 10) or was untraceable (*n* = 2). The ages at which critical events occurred and cause of death where known, are presented in Table 1; ages are aggregated into 5-year bands and the ages of 12 individuals still alive in 2013 are not shown, to prevent re-identification. For the two untraceable individuals, ages at last known clinical contact (1959) are shown in Table 1. Because it is unknown whether these two individuals died between 1959 and 2013, and if so when, they were excluded from the analysis. We discuss below how lack of information about their dates and causes of death might have affected the study.

**Table 1:** Outcome in 12 individuals with Apert syndrome who were living in 1959 and censored before study closure in 2013.

Age range (years)	Untraceable after 1959	Died	Cause of death
5–9		1	respiratory obstruction
10–14			
15–19	1		
20–24			
25–29		1	pneumonia and epilepsy
30–34		1	acute myeloid leukaemia
35–39		1	carcinoma of ovary
40–44			
45–49	1		
50–54		1	pneumonia
55–59		1	carcinoma of breast
60–64			
65–69			
70–74		2	cor pulmonale; congestive cardiac failure
75–79		2	carcinoma of lung; cerebrovascular accident

**Fig. 2:**
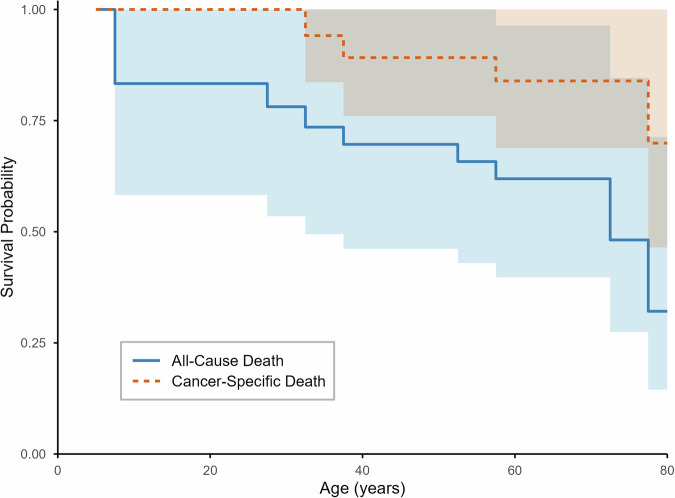
Kaplan-Meier survival curves in Apert syndrome. The dashed red and solid blue lines show Kaplan-Meier survival plots, by 5-year age bands, for cancer-specific and all-cause mortality, respectively. The coloured shaded regions around each line indicate the corresponding 95% confidence intervals.

Table 1 shows that there were four deaths from cancer during the follow-up period (1959–2013), which included two (one each with acute myeloid leukaemia and ovarian carcinoma) in younger individuals aged between 30 and 40 years (yr) at the time of death. Each of the four cancers involved a different combination of organ and tissue origin, which in turn differed from those previously reported in single cases of tumours in children with Apert syndrome, comprising rhabdomyosarcoma [22], bladder cancer [23], ovarian dysgerminoma [24] and medulloblastoma [25]. Aside from malignancy, six other causes of death (infective, cardiorespiratory or neurological) were recorded (Table 1); in two of these deaths, which occurred in the age groups 5–9 yr and 25–29 yr, it is possible that the Apert syndrome could have contributed causally to mortality, but no details are available. The standardised risk ratios and Kaplan-Meier plots for cancer mortality and all-causes mortality are presented in Table 2 and Fig. 2, respectively. Overall, the data are consistent with no excess cancer-specific or all-cause mortality, but given the small numbers and consequent limited statistical power of the study, mortality rates in Apert syndrome could plausibly be up to around 3 times greater both for cancer and for all causes, compared to population background.

**Table 2:** Standardised mortality ratios for individuals with Apert syndrome compared to the general population of England and Wales.

Cause of death	Expected number of deaths	Observed number of deaths	Standardised mortality ratio (95% CI)
Cancer	3.14	4	1.27 (0.35–3.26)
All causes	6.42	10	1.56 (0.75–2.86)

*CI* confidence interval.

Overall, based on these data, we find no evidence to support the hypothesis that Apert syndrome confers a high risk of developing specific types of cancers, compared to the population as a whole. None of the individuals with cancer was aged under 30 yr, providing no support for a predisposition to cancers during childhood or early adulthood as raised by the previous case reports [22–25]; this was also the conclusion of the study of Tovetjärn et al., which identified no deaths or cancers in 33 individuals with Apert syndrome followed up to age range 18–49 years [17]. Evidence is similarly lacking for an increase in cancers associated with Crouzon and Pfeiffer syndromes (allelic craniosynostosis disorders caused by different mutations in *FGFR2*, several of which are also recorded as somatic mutations in cancer [27–29]), although we are unaware of any comparable survival studies in those disorders. Mirroring our findings, in a large follow-up study of achondroplasia (caused by weakly activating germline mutations in the related *FGFR3* gene that, when arising somatically, have been identified in bladder cancers [29]), deaths from malignant neoplasms appeared reduced compared to the population average, although the difference was not statistically significant [33]. These observations for *FGFR2* and *FGFR3* contrast with Costello and Noonan syndromes associated with activating mutations in *HRAS* and *PTPN11* respectively, RASopathies that are associated with increased risk of early onset cancers [34–37].

The resolution to the paradox that some heterozygous mutations confer little or no increased cancer risk when the corresponding somatic mutations are clearly oncogenic, may lie in the role played by cell competition in the origins of neoplasia. This describes the situation whereby cells with different genetic constitutions interact competitively, and this can lead to death of viable wild type cells, if they are sensed as less fit than their mutant neighbours (reviewed in [38, 39]). This situation does not apply in constitutionally heterozygous individuals, because all mutant cells are genetically equivalent. Cancer predisposition could nevertheless occur (as in RASopathy mutations) if secondary somatic mutations [40] or epigenetic changes synergise functionally with the constitutional mutation [41]; presumably this occurs rarely in the case of the Apert syndrome *FGFR2* mutations.

Whilst this study provides unique information on Apert syndrome, its small size (*n* = 24) confers important limitations to its statistical power. For example, information about the deaths of the two untraceable individuals could affect our findings. The most extreme circumstance, resulting in the largest increase to the standardised mortality ratios, would be if they had both died in 1959, at the very start of the study, effectively contributing no time at risk, and in particular if both had died from cancer. Making this extreme assumption as a sensitivity analysis, the standardised mortality ratios and confidence bounds would increase (to 1.91, 95% CI: 0.70–4.16, for cancer-specific, and 1.87, 95% CI: 0.97–3.27, for all-cause mortality), but these figures do not alter the qualitative conclusions from the main analysis.

These issues notwithstanding, potentially the most impactful finding from this study is that over half the cohort of individuals with Apert syndrome survived at least to the age of 60 years; moreover there were no additional cancer registrations in those surviving to the end of the study period. This knowledge will be important to parents and patients, who may be concerned about the long-term health implications of a diagnosis of Apert syndrome. Our attempt to gather data on adult health outcomes had limited success, because we only obtained permission to visit 5 subjects. However, this did help to validate the study by proving that all five individuals indeed had Apert syndrome. Of note, this study could not have been performed systematically by analysis of death certificates mentioning a diagnosis of Apert syndrome, because only one of the 10 certificates did so (another mentioned multiple congenital handicap, the remaining eight made no reference to any underlying syndrome).

Of the five individuals whom we were able to visit, three (two with *FGFR2* NM_000141.5:c.755 C > G and one with NM_000141.5:c.758 C > G) had been able to live independently and had been in employment for many years, whereas the other two were long-term unemployed and dependent. Only two of the five had received any craniofacial surgery during childhood (at the ages of 1 and 11 years); the surgical, paediatric and educational approach to the management of Apert syndrome has been transformed since the birth of this cohort (which occurred on average over 80 years ago), so it is anticipated that the functional outcome for a contemporary cohort would be considerably improved compared to the group presented here.

## Data Availability

Access to data is subject to a Data Sharing Agreement with NHS Digital (ref. DARS-NIC-148106-PP9LS-v.4.4). Parties interested in accessing the primary data should contact the authors in the first instance.
